# Characterizing cell interactions at scale with made-to-order droplet ensembles (MODEs)

**DOI:** 10.1073/pnas.2110867119

**Published:** 2022-01-24

**Authors:** Justin L. Madrigal, Nathan G. Schoepp, Linfeng Xu, Codian S. Powell, Cyrille L. Delley, Christian A. Siltanen, Jay Danao, Maithreyan Srinivasan, Russell H. Cole, Adam R. Abate

**Affiliations:** ^a^Scribe Biosciences, San Francisco, CA 94107;; ^b^Department of Bioengineering and Therapeutic Sciences, California Institute for Quantitative Biosciences, University of California, San Francisco, CA 94158;; ^c^Chimera Bioengineering, South San Francisco, CA 94080;; ^d^Chan Zuckerberg Biohub, San Francisco, CA 94158

**Keywords:** droplet microfluidics, cell–cell interaction, single-cell analysis, functional sorting, cell therapy

## Abstract

Cell–cell interactions are important to numerous biological systems, including tissue microenvironments, the immune system, and cancer. However, current methods for studying cell combinations and interactions are limited in scalability, allowing just hundreds to thousands of multicell assays per experiment; this limited throughput makes it difficult to characterize interactions at biologically relevant scales. Here, we describe a paradigm in cell interaction profiling that allows accurate grouping of cells and characterization of their interactions for tens to hundreds of thousands of combinations. Our approach leverages high-throughput droplet microfluidics to construct multicellular combinations in a deterministic process that allows inclusion of programmed reagent mixtures and beads. The combination droplets are compatible with common manipulation and measurement techniques, including imaging, barcode-based genomics, and sorting. We demonstrate the approach by using it to enrich for chimeric antigen receptor (CAR)-T cells that activate upon incubation with target cells, a bottleneck in the therapeutic T cell engineering pipeline. The speed and control of our approach should enable valuable cell interaction studies.

## Significance

The study of specific cell–cell interactions at scale would be a significant advancement in single-cell biology with clear utility in immuno-oncology. Our development of Droplet Assembly provides a tool for such studies by extending the benefits of single-cell droplet microfluidics to high-order cell analyses. This technology allows for the construction, sorting, and downstream processing of cell–cell interactions and is compatible with single-cell genomic readouts.

The immune system relies on a massive number of individual cell–cell interaction events. These interactions are key to distinguishing self from nonself and foundational to emerging cancer immunotherapies such as immune checkpoint blockade, adoptive T cell therapy, and cancer vaccines (1). Nevertheless, despite the centrality of cell interactions in biology, few technologies are available for characterizing them at scale. Instead, interactions are usually deciphered from bulk cultures based on cytokine release, cytotoxicity, surface marker presentation, or single-cell transcriptional profiling (2, 3). While useful for characterizing synergistic behavior between cell types and overall response, bulk data lacks the detail necessary for precisely mapping cellular interactions at the heart of many biological systems, especially the immune system (4).

Characterizing cell–cell interactions is thus a large and unmapped frontier that has the potential to impact the treatment of numerous health maladies, especially cancer and autoimmunity (5). Although this has motivated new technologies, isolating specific interactions at scale remains difficult. For example, microfluidic approaches with droplets, microchambers, and wells exploit picoliter volumes to quantitate secreted cytokines and barcoding strategies to analyze DNA, messenger RNA, and proteins (6, 7). By leveraging the inherent throughput of these methods, tens of thousands of cells can be analyzed, providing rich data. However, these approaches do not control cell loading, yielding mostly empty compartments. While this inefficiency is acceptable for single-cell studies, cell–cell interactions require combinations, which are impractical to generate randomly. For example, with a common loading of ∼5%, high-throughput approaches with 100,000 microcompartments capture ∼5,000 single cells but just ∼100 random cell–cell pairs. By implementing controlled cell loading with microfluidic cell pairing (8), printed droplets (9), or light-induced dielectrophoresis (10), every compartment can contain the needed number of specific cells, greatly increasing efficiency. These approaches perform well within their intended usages and function to analyze thousands of pairs. However, they were not designed with the intention of profiling the complexity of systems such as the immune repertoire, which comprises millions of T cells (11). Thus, there remains a need for technologies capable of rapidly generating controlled cell combinations; such an approach would open the way for characterizing cellular interactions at scale and be valuable for numerous studies across fields including cancer, immunology, and microbiology (12–14).

In this paper, we describe a high-throughput technology for cell interaction profiling. Our core innovation is the integration of dielectrophoretic droplet sorting with deterministic merging to build multicomponent droplets containing defined combinations of reagents, beads, and cells. These made-to-order droplet ensembles (MODEs) can currently be generated at >10 Hz and subjected to detailed chemical, microscopic, and genomic analysis, making them useful for cell interaction studies. To demonstrate the utility of our approach, we profile chimeric antigen receptor (CAR)-T and CD19-presenting cell interactions using cytokine capture beads to quantitate activation via interferon-gamma (IFN-γ) secretion. Using this assay, we enrich for CAR-T cells that activate upon incubation with cancer cells and identify the associated gene expression. The speed, robustness, and simplicity of MODEs make large-scale cell interaction studies more feasible and can potentially play an important role in the study of the immune system.

## Results

### Droplet Assembly for Studying Cell–Cell Interactions.

**Fig. 1.**
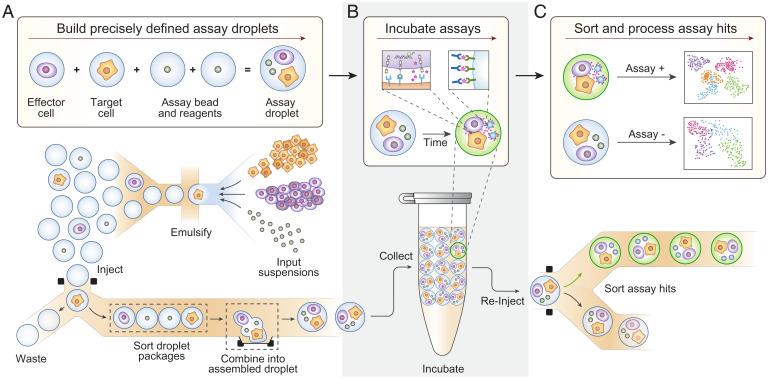
The Droplet Assembly workflow allows for precisely defined assay droplets to be built, incubated, and sorted based on phenotypic readout (cytokine secretion). (*A*) Droplet assembly deterministically combines single-cell–containing droplets and reagent-containing droplets to create thousands of identical, defined assay droplets. (*B*) Assay droplets are incubated for a defined period, and secreted cytokine is captured onto the surface of a detection bead. (*C*) Assay droplets are sorted for downstream processing based on the presence of cytokine.

Droplet microfluidics allows high-throughput characterization with chemical, proteomic, and genomic readouts but struggles to extend this utility beyond single-cell analysis. For example, assaying effector–target interactions requires, at a minimum, that two cells and assay components be combined in the same droplet (Fig. 1 *A*, *Upper*), but relying on random encapsulation to create such ordered groupings is prohibitively inefficient (15). MODE assembly overcomes this inefficiency by building multicomponent droplets in a stepwise, deterministic process that is fast and flexible (Fig. 1 *A*, *Lower*). Aside from their defined composition, MODE droplets are identical to ones formed by other methods (16) and, accordingly, can be subjected to common manipulations, such as incubation to allow cell–cell interactions to occur (Fig. 1*B*), fluorescent analysis, sorting, and sequencing to characterize interaction behavior (Fig. 1*C*).

**Fig. 2.**
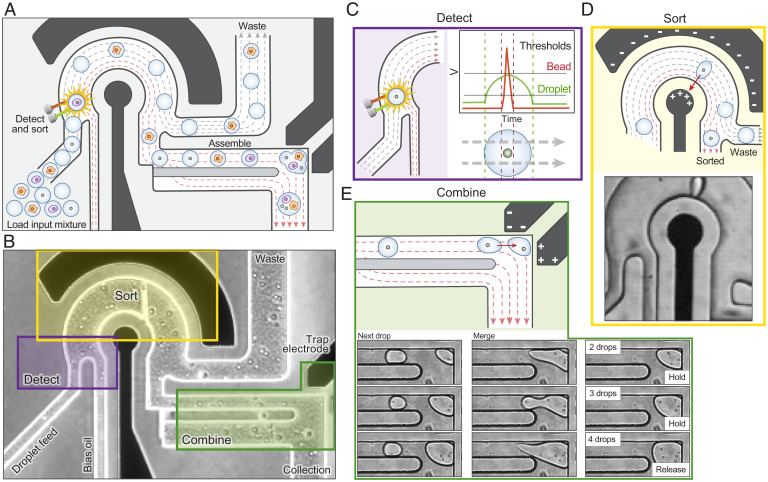
Droplet assembly combines a fluorescent droplet sorter and a novel merging trap to selectively detect, sort, and combine desired input droplets to build defined assay droplets. (*A*) Schematic representation of the microfluidic chip. Black regions represent electrodes, and dashed lines flow. (*B*) Phase contrast image of the microfluidic chip showing detection, sorting, and combining regions. Droplet assembly builds on the detection (*C*) and sorting (*D*) of traditional fluorescent droplet sorters through the incorporation of a dielectrophoretic trap (*E*) that selectively pulls droplets out of flow and merges them into the assembling MODE.

The key to the efficiency of MODE assembly is a mechanism by which droplets of specific composition are combined into the final desired multicomponent droplet. We accomplish this by selecting and merging single-component droplets from a mixed emulsion using dielectrophoretic sorting (Fig. 2*A*). Sorting has several advantages for delivering droplets on demand, including the ability to select from droplets of distinct composition that would be incompatible in a single aqueous stream, such as miscible reagents; the ability to use laser-induced fluorescence to interrogate droplet contents for labels, beads, and cells and to exclude ones with improper contents; and extreme speed, capable of sorting up to 30 kHz (17). For the sorter to deliver specific droplets on demand, their contents must thus be detectable by fluorescence, requiring dye labels (for reagents) or staining (for cells, beads). Thus, the first step in the MODE workflow is to generate the mixed emulsion by labeling the discrete and reagent components as needed, separately emulsifying each, and combining the droplets. The mixed emulsion is then introduced into the assembly device, where the droplets are scanned, sorted, and combined according to the desired MODE composition (Fig. 2 *A* and *B*). Optical scanning captures fluorescence signals from the droplets (Fig. 2*C*), used by the sorter to decide whether to deliver the given droplet to the MODE. If a droplet is to be added, the sorter activates the field, applying a dielectrophoretic force that deflects it (Fig. 2*D*) into the combiner (Fig. 2*E*). In the combiner, a bipolar electrode generates a field that captures sorted droplets, overcoming the shear-induced flow of the carrier oil and halting the droplet. As additional droplets are added, they collide and coalesce with previously trapped droplets due to the electric field (Fig. 2 *E*, *Lower*). This cycle repeats until all droplets are combined for the desired MODE, upon which the trapping field is deactivated, and the finished MODE released into the collection tube (Movie S1). The next MODE then begins assembly.

Constraints in the size and constituency of MODEs are governed by chip architecture. For example, the geometry of the droplet trap dictates its volume and limits the number of droplets that can be added per MODE. The current chip is designed to sort input droplets of 40 to 60 pL (42- to 49-µm diameter). We tested the capacity of this trap design to combine droplets and found that 300 pL MODEs, corresponding to six input droplets, were reliably generated, with few MODEs successfully incorporating more than eight input droplets (*SI Appendix*, Fig. S1 *A* and *B*). These limitations on droplet input number and output volume can be easily changed by adjusting chip dimensions.

A unique and valuable property of MODE for generating cell combinations is its superior speed, which is influenced by three factors: the time between required droplets, the time for a sorted droplet to reach the combiner, and the time for an assembled MODE to release. The time between required droplets is tied to sorting speed and the proportion of that droplet type in the single component emulsion, λ. To yield high proportions of single-cell occupancy λ is typically kept below 15% during encapsulation. For a conservative sorting rate of ∼300 Hz and loading of 5%, there is an average wait time of ∼70 ms between required droplets. Because droplet delivery is order agnostic, adding additional components to the mixed emulsion, like different cell types, beads, or reagents, reduces λ proportionally; for example, a two-component emulsion loaded at 5% each has a net λ for each component of 2.5% in the mixed emulsion, corresponding to a wait time of ∼140 ms between required droplets. The time for the sorted droplet to reach the trap is ∼25 ms, with ∼25 ms for the MODE to release from the trap, yielding an overall assembly speed for a two-component droplet of ∼5 Hz or ∼18,000 per hour. Speed can be increased by concentrating the mixed emulsion to reduce wait time at the expense of increased multiple cell and bead encapsulations that reduce the composition accuracy of the MODEs. Alternatively, speed increases proportionally with sorting rate, affording a potential ∼100-fold increase by implementing higher-throughput methods (30 KHz) that require enhanced optics to scan the faster droplets (18) and would allow production of over a million MODEs per hour. Currently, we have achieved assembly rates of up to 12 Hz for two-component MODEs, allowing the production of 100,000 MODES in just over 2 h.

### Precision Droplet Assembly.

**Fig. 3.**
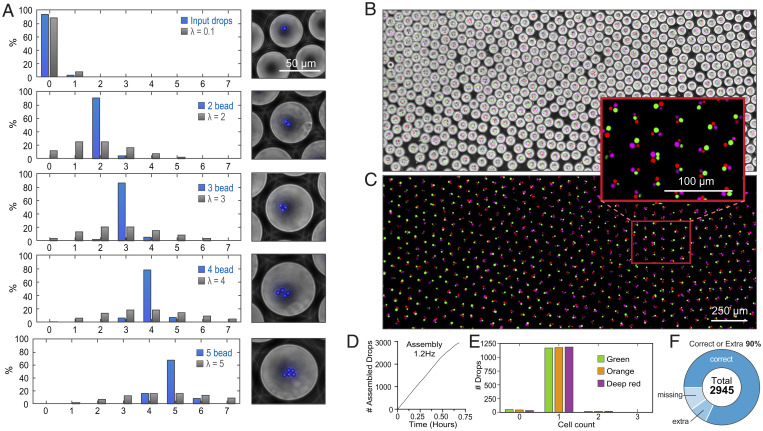
Droplet assembly bypasses Poisson sampling limitations for the construction of complex droplets. (*A*) Assembled droplets containing two to five fluorescent beads were constructed, and the resulting content distributions were compared to theoretical Poisson distributions. Composite (*B*) and fluorescent (*C*) images of assembled droplets combining one orange-stained, one green-stained, and one deep red–stained RAJI cell. (*D*) Run chart of droplet assembly. (*E*) One-dimensional distributions of assembled three-color RAJI droplets. (*F*) Pie graph showing percentages of correctly built droplets, droplets containing extra components, and droplets with missing components.

To assess the effectiveness of our approach, we use it to generate MODEs containing defined numbers of fluorescent beads (Fig. 3*A*). Beginning with an input emulsion of sparsely loaded beads (λ = 4%), we create four output emulsions comprising 1,000 MODEs programmed to contain different numbers of beads (1- to 2-Hz assembly). We assess the distribution of beads per MODE by fluorescence microscopy and determine content accuracies of 92 ± 2.1, 88 ± 1.0, 78 ± 3.1, and 75 ± 7.2% for droplets programmed with two, three, four, or five beads, respectively, over two separate runs (Fig. 3*A*). Efficiency reduces as beads are added due to increased sampling of sorting events and trapping errors, which become more frequent as MODE volume increases. Sorting errors are similar to those found in previous droplet-sorting studies (19), and failures stem from the misreading of droplet contents because of the overloading of input droplets or missorts because of flow interference from dust or occasional merged droplets in the mixed emulsion (20). Trapping errors can include early MODE release, failure to release, and incomplete merging (*SI Appendix*, Fig. S1 *A* and *B*). Nevertheless, pairwise loading efficiency is high, and even at five beads, the device achieves ∼75% efficiency, which is over a billion times more efficient than random loading from a sparse input suspension.

In a combinatorial assay comprising cells of different types, MODE assembly must distribute the correct number of each cell into the combined droplets, which requires that the different cell types be distinguishable. To illustrate this capability, we introduce a sparsely loaded emulsion (λ = 7.5%) of green, orange, and red fluorescently stained Raji cells and use the combiner to build 3,000 MODEs containing exactly one of each cell color (1.2-Hz assembly) (Fig. 3 *B*–*D*). We determine ∼82% are correctly assembled, with ∼10% missing a cell and 8% containing an extra cell (Fig. 3 *E* and *F*). Importantly, there are negligible effects on cell viability throughout the process of droplet assembly (*SI Appendix*, Fig. S2).To illustrate the scalability of the approach, we perform an additional experiment in which we generate 100,000 MODEs containing a red and blue bead (input emulsion λ = 18%, 5.5-Hz assembly), achieving a construction accuracy of 80% for exactly one of each color and 95% of droplets containing at least one of each color (*SI Appendix*, Fig. S3). Pairwise combinations are useful for T cell activation studies but, presently, can only be made in the low thousands using costly instrumentation (21).

### Characterizing CAR-T Activation at Scale.

The principle of CAR-T cell therapy is to modify the body’s natural T cells to express an engineered receptor that specifically recognizes the cancer cells upon which the T cells activate and generate an immune response that can, in some instances, eradicate the disease (22). An important part of CAR-T engineering is to identify CAR-Ts that specifically and efficiently kill cancer cells. Bulk approaches for accomplishing this, however, lack the single-cell resolution required to identify CAR-Ts with desired properties and are costly, requiring multiple rounds of testing and screening. By contrast, MODE assembly provides a route to efficiently identify these cells by individually measuring their activation when incubated with cancer cells.

**Fig. 4.**
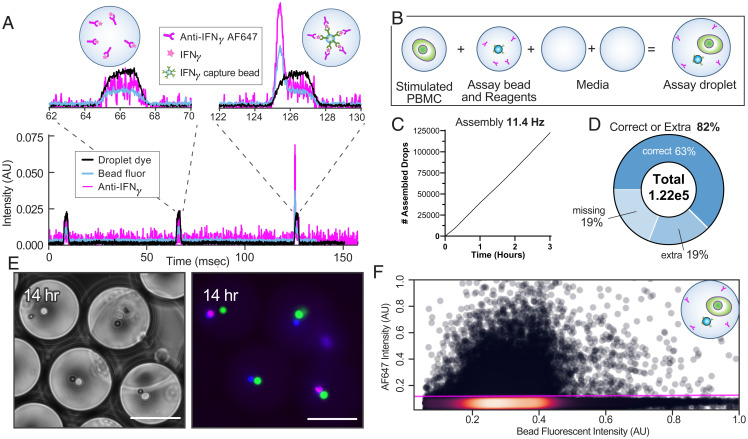
IFN-γ detection assays can be assembled for individual cell secretion events at scale. (*A*) Timetrace of fluorescent droplet detection showing positive signal from 1 nM IFN-γ standard. (*B*) Stimulated PBMC, assay detection beads, assay detection reagents, and media are combined into assay MODEs. (*C*) Run chart for MODE assembly. (*D*) Over 122,000 MODES were assembled with 82% containing all required assay parts. (*E*) Representative composite fluorescent images of assay MODES at 14 h. (Scale bars, 50 µm.) (*F*) Assay MODES were reinjected, and the percentage of MODES above a threshold for IFN-γ was determined.

To identify activated cells, we first build MODEs containing a fluorescence relocation assay for secreted interferon-γ (IFN-γ). Detection beads coated with a capture antibody localize IFN-γ, which is bound by a fluorescent secondary antibody to produce a concentrated and sortable signal (Fig. 4*A*) (23, 24). We tested the utility and throughput of this approach using artificially stimulated peripheral blood mononuclear cells (PBMCs) (Fig. 4*B*). Using MODE assembly, we construct 122,731 droplets containing a phorbol 12-myristate 13-acetate (PMA)/ionomycin-activated PBMC, achieving a complete assay accuracy of 82% (Fig. 4 *C* and *D*) (input emulsion λ = 7.3%, 11.4-Hz assembly). Following a 14-h incubation (Fig. 4*E*), the MODE emulsion was reinjected into droplet sorting, revealing 17.9% hits within the emulsion (Fig. 4*F*), which agrees with previous analyses of PBMC IFN-γ secretion following PMA/ionomycin stimulation (25). We next apply the approach to detect activation of a mixture of CD4+ and CD8+ αCD-19 CAR-T cells, again stimulated with PMA/ionomycin. A total of 7,000 assembled MODEs (*SI Appendix*, Fig. S4*A*) (input emulsion λ = 7.7%, 1.4-Hz assembly) were generated and incubated for 12 h to allow for T cell activation, IFN-γ secretion, and pulldown onto the bead (*SI Appendix*, Fig. S4*B*). MODEs were then interrogated with the droplet sorter to quantify IFN-γ–positive droplets (*SI Appendix*, Fig. S4*C*). Of the interrogated droplets, 34% were positive for IFN-γ. Given that IFN-γ production is expected predominantly from CD8^+^ T cells, the result falls within the range expected for typical CD4/CD8 ratios and demonstrates the effectiveness of the assay (26, 27).

**Fig. 5.**
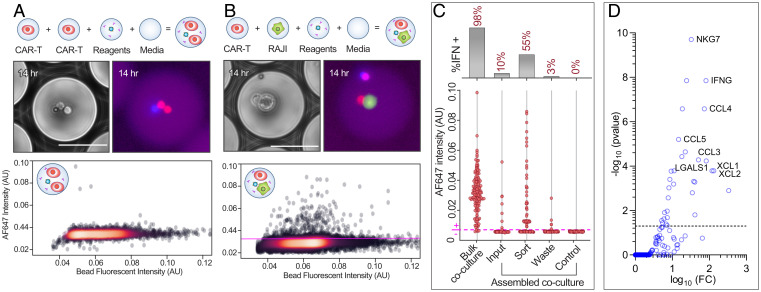
CAR-T cell–cell interactions can be assayed and enriched for activation using the droplet assembly workflow. Two sets of cell pairs, CAR-T and CAR-T (*A*) or CAR-T and RAJI (*B*), were assembled into IFN-γ assay droplets and allowed to incubate for 14 h to allow for activation and IFN-γ secretion, and the emulsion was sorted for drops above the scatter plot threshold (magenta). (Scale bars, 50 µm.) (*C*) Detection beads collected from broken emulsion samples, and bulk coculture samples were quantified for signal intensity by fluorescent microscopy, and beads above the control intensity (magenta) were counted as assay positive. (*D*) Up-regulated genes for GZMB overexpressing cells in the sort compared to waste. (Off-axis: GZMB, log10FC = 4.71, −log10(pvalue) = 35.80).

With the assay validated, we next apply it to enrich for CAR-T cells capable of activating upon interaction with a cancer cell. Enrichment of specific CAR-T cells is currently a bottleneck in CAR-T engineering. We assemble 16,000 MODEs (input emulsion λ = 13%, 3.1-Hz assembly) containing a CAR-T cell, Raji cell, IFN-γ bead, and assay reagents. Separately, as a negative control, we prepare 10,000 MODEs with a homotypic pairing of two CAR-T cells and assay reagents but no Raji cell. Upon incubation for 14 h, we identify activated cells in the CAR-T plus Raji MODEs but not the homotypic controls (Fig. 5 *A* and *B*). To enrich for activated cells, we sort the emulsion based on a fixed fluorescence threshold (Fig. 5*B*) collecting the positive and negative outputs in separate tubes. We recover the contents of the samples via demulsification (28) and analyze the fluorescence intensities of the beads, identifying a 5.5-fold enrichment for a positive signal relative to the unsorted input emulsion and an 18.3-fold enrichment relative to the waste stream (Fig. 5*C*).

To uncover the molecular underpinnings of the activation behavior, we repeated the MODE assay assembly (*SI Appendix*, Fig. S5) and performed single-cell RNA sequencing of the collected sort and waste samples. We first compare overall gene expression between the two unfiltered populations. We find that granzyme B (GZMB), a serine protease associated with activated CD8+ cytotoxic T cells, was significantly up-regulated in the sorted population (29). Moreover, after filtering data to remove barcodes containing a low number of unique molecular identifiers, we show that the percentage of CD8+ cells expressing GZMB is higher in the sorted versus the waste populations (*SI Appendix*, Fig. S6). This small enrichment corresponds to cells that were above our threshold of IFN-γ detection at the time of sorting and high in GZMB expression during sequencing preparation. Variability in the ratio of IFN-γ to GZMB expression is to be expected (30, 31). Using GZMB as a marker of activation, we compare gene expression in cells expressing GZMB (log_2_ count ≥2) in the sorted and waste populations to cells in the waste population that showed no expression of GZMB (i.e., background gene expression) (Fig. 5*D*). In the sorted GZMB-expressing subpopulations, we observe several markers of activation (*SI Appendix*, Table S1) (32–36).

## Discussion

Studying cell–cell interactions at scale will impact our understanding of fundamental biological processes and disease, especially involving the immune system. However, current technologies lack the speed and precision to generate combinations with the throughput needed to characterize systems comprising millions of genetically and phenotypically distinct cells. MODE assembly can currently produce 100,000 cell interaction assays in under 3 h, and with improvements in sorting and trapping speeds, this rate can increase further. MODE assembly thus provides an important step forward in cell interaction profiling, breaking through longstanding barriers in the precision with which specific cell types can be combined, the sophistication with which the assay environment can be engineered and the scale at which these multicellular assays can be performed. The key innovation that enables these breakthrough capabilities is deterministic cell assembly, rather than random grouping, to build multicellular microenvironments, increasing efficiency by hundreds for pairs and billions for quintets. Importantly, and as we have shown, the assembled MODEs are otherwise identical to common water-in-oil droplets and, thus, can leverage the innovations of this field from the last two decades, including the ability to characterize interaction behavior with powerful readouts using optical, chemical, and barcode-based genomic and proteomic methods and to manipulate and sort them for large-scale cell interaction screens. While we have provided a demonstration for human immune cell profiling, MODE assembly should be applicable to other cell types, including stem and cancer cells useful for generating hybridomas, spheroids, and organoids. Furthermore, alternative assays to cytokine secretion should be easily integrated into MODEs including cell-mediated cytotoxicity and antibody production/specificity. Similar water-in-oil droplets are also compatible with nonmammalian systems, including yeast, bacteria, and archaea, and thus, MODE assembly should be useful for studying microbial consortia important to the microbiome and function of biocircuits and molecular biosynthesis.

## Materials and Methods

Input droplets for MODE assembly are first generated using a flow-focusing droplet generator. Each component is individually emulsified, and the emulsions are mixed in a single syringe. Emulsions and oil are loaded into syringes, attached to the microfluidic chip using small bore polyethylene tubing (PE-2, Scientific Commodities, Inc.), and mounted on syringe pumps (New Era). Sort and waste channels are attached to the microfluidics using small bore polyether ether ketone tubing (IDEX). A custom LabView application (JKI) is used to initialize and carry out an assembly run. Droplet fluorescence data are acquired by a field programmable gate array (FPGA; National Instruments). All droplets are identified by the peak and width of fluorescent droplet dyes included in every droplet. Fluorescent peaks occurring within the window of droplet detection distinguish the contents of a given droplet. Cells are prestained with green and deep-red dyes (CellTracker, Thermo Fisher). Fluorescent beads used in experiments were purchased with blue and red dye signatures (Spherotech). Sorting gates are set within the application to identify droplets of interest and the number of each droplet type required per MODE, and the total number of MODEs to be built are specified. The FPGA controls signals sent to two electrodes via high-voltage amplifiers (2,220; Trek). The sorting electrode is triggered when droplets missing from an assembling MODE are identified. The trap electrode signal is turned off once all components of the MODE have been sorted and merged. The output MODE emulsion is either directly collected within imaging chambers for packed droplet imaging, or it is collected within a larger emulsion of empty droplets for incubation.

## Supplementary Material

Supplementary File

Supplementary File

## Data Availability

Sequencing data have been deposited in NCBI Gene Expression Omnibus (accession no. GSE193197). All other study data are included in the article and/or supporting information.
