# Supplementary figures and images for: Characterizing cell interactions at scale with made-to-order droplet ensembles (MODEs)
Source: Proc Natl Acad Sci U S A. 2022 Jan 24;119(5):e2110867119. doi: 10.1073/pnas.2110867119 (PMC8812558; doi:10.1073/pnas.2110867119)

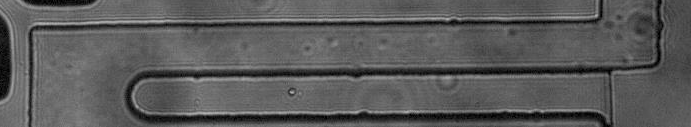

Supplement: Supplementary File [file pnas.2110867119.s01.gif]
