# Supplementary material for: Characterizing cell interactions at scale with made-to-order droplet ensembles (MODEs)
Source: Proc Natl Acad Sci U S A. 2022 Jan 24;119(5):e2110867119. doi: 10.1073/pnas.2110867119 (PMC8812558; doi:10.1073/pnas.2110867119)
Supplement: Supplementary File [file pnas.2110867119.sapp.pdf]

Supplementary Information

SI Materials and Methods

Device Fabrication

Droplet assembly chips are fabricated from poly(dimethylsiloxane) (PDMS) using standard soft lithography techniques from photopatterned multilayer molds. The master molds are created from two sequential layers of SU8 photoresist spun onto a silicon wafer. The wafer is developed to remove unpolymerized photoresist, and uncured PDMS (10:1 polymer to cross-linker ratio) is poured over the photopatterned mold and cured in an oven at 80 °C for 80 min. The PDMS molds are extracted from the masters with a scalpel and punched with access ports using a 0.75-mm biopsy core, then plasma bonded to 2″ × 3″ glass slides. Fluidic channels are rendered hydrophobic by flushing with Aquapel, which is then removed by baking at 80 °C for 10 min. Electrode channels are filled using syringes containing a liquid metal alloy of Gallium, Indium and Tin and interfaced with high-voltage amplifiers (Trek Model 2200).

Optical Configuration

Excitation light is provided using a 4-line (405, 488, 561,632 nm) laser platform (Hubner Photonics) and focused onto droplets using a 10x objective. Emitted light is collected and filtered by a quad-bandpass (Semrock), and redirected by a series of dichroic filters (Semrock) to detection PMTs (Hamamatsu H10722-20) with bandpass filters providing detection channels centered at 405, 510, 571, and 697 nm. Infrared LED light is used to illuminate the microfluidic chip for imaging and redirected to a FLIR Blackfly® USB camera.

Generation of anti CD19 CAR T Cells

The pCD19 lentiviral transfer vector used in this study was constructed in the pCDH backbone (SBI, CD813A-1). Briefly, the pCDH insert sequences between XhoI/NotI were removed and replaced with an insert containing (5'→3') the human PGK promoter, a TISU element (Elfakess et al., 2011), mCherry, a T2A peptide and an anti-CD19 CAR. The anti-CD19 CAR transgene is comprised of a CD8α signal peptide, followed by an N-terminal myc tag fused to an anti-human CD19 scFv (clone FMC-63), a CD8α hinge and transmembrane domain, with CD137 costimulatory and CD3ζ endodomains (Milone et al., 2009). CAR T cells were generated using a 2nd generation lentiviral system comprised of the pCD19 CAR transfer vector pMD2.G (Addgene #12259), and psPax2 (Addgene #12260). LX293T cells (Clontech, Cat#632180) plated at 5 x 105 cells/well in a 6-well plate for 24 hours were transfected with the plasmids using Fugene (Promega, Cat#E2311). Viral supernatants were harvested 48 hours after plasmid transfection, and cellular debris were removed by centrifugation at 400 x g for 5 minutes.

Primary human T lymphocytes (CD3+) were purified with RosetteSep (StemCell Technologies 15021) from peripheral blood obtained from anonymous healthy donors. Primary CD3+ cells were activated for 24 hours with 1:1 CD3/CD28 beads to cell ratio (Gibco/Thermo Fisher Cat#11131D) in T cell Culture Media (X-Vivo 15 Lonza 04-418Q with 5% human AB serum Gemini Bio 100-512, 10mM N-acetyl-cysteine Milipore Sigma A9165 neutralized with 0.9 molar equivalents of NaOH Milipore Sigma 100-512, and 55uM 2-Mercaptoethanol ThermoFisher 21985023) supplemented with 66 IU recombinant IL-2/ml (Peprotech AF-200-02). CD3+ cells at 1 x106 cells/ml were transduced with lentivirus at a 1:1 mixture (T cells in Culture Media: cleared lentivirus supernatant) for 24 hours. The transduced T cells were pelleted by centrifugation at 400 x g for 5 minutes, the lentiviral supernatant was removed by aspiration, and the T cell pellet was resuspended in T cell Culture Media. 96h post-transduction, the Dynabeads were removed and the T cells were expanded and maintained at 2 x 106 T cells/ml in T cell Culture Media supplemented with 66 IU/ml of recombinant IL-2 every 2-3 days.

The transduction rate was determined by flow cytometry on a Cytek Aurora for mCherry on live and single cells. Briefly, T cell samples were collected and transferred to 12x75 mm polypropylene tubes. Cells were

washed with PBS and resuspended to 5-10 x 106 cells/ml in PBS with 0.5ug/ml Propidium Iodide (ThermoFisher P1304MP) for live cell determination. At least 10,000 cells were analyzed. Data analysis was performed using Cytobank, and untransduced T cells were used to set the mCherry negative gate.

Cellular Culture

CAR-T cells were cultured in 75-cm^2 flasks in X-Vivo^{TM} 15 Serum-free Hematopoietic Cell Medium (Lonza BE02-060Q) supplemented with IL-2 (Peprotech cat. 200-02) at 33-66 units/mL. RAJI (ATCC CCL-86^{TM}) and PBMC (StemCell cat. # 70025.1) were cultured in 75-cm^2 flasks in RPMI-1640 medium (ATCC 30-2001^{TM}) supplemented with 5% FBS and antibiotics. Prior to assembly experiments cells were fluorescently stained for easy detection. One million cells were stained with 0.5 µM Cell-Tracker Green CMFDA, 0.5 µM Cell-Tracker Orange, or 0.25 µM Cell-Tracker Deep Red (Thermo Fisher) for 10 minutes at 37°C. The cells were washed twice in PBS and resuspended directly into droplet medium.

Assay Bead synthesis

IFN-γ capture beads were prepared using N-hydroxysuccinimide (NHS)/1-ethyl-3-(3-dimethylaminopropyl) carbodiimide hydrochloride (EDC) chemistry. 5.5 um carboxyl-functionalized polystyrene beads (Spherotech catalog no. CFP-5045-2) were washed and activated using 45 mM NHS and 20 mM EDC in 50 mM 2-morpholinoethanesulfonic acid monohydrate (MES) + 150 mM NaCl pH 6.0. Beads were then washed and resuspended in PBS. Conjugation was performed in PBS by incubating activated beads and IFN-γ capture antibody (R&D Systems catalog no. DY285B) for 2 hrs at room temperature at ~5e7 beads/mL and 1.0 mg/mL antibody. The reaction was quenched by incubating with 50 mM Tris-HCl pH 8.5 for 15 min. Beads were then washed and resuspended in PBS. All washes were performed by filtering beads using pre-washed 0.2 um cellulose-acetate filters (Sigma catalog no. CLS8160) via centrifugation at 1000 rcf for 30 sec.

Droplet generation for MODE assembly

To generate droplets for combinatorial tests, fluorescent beads (Spherotech) or fluorescently stained RAJI cells were suspended at 3e6/mL in X-Vivo 15^{TM} along with fluorescent-modified 10 kDa dextran as encoding dye (Thermo Fisher). Droplets were generated using a flow-focusing droplet generator chip with a 4-way droplet splitter downstream of initial droplet formation. In studies utilizing dummy drops to help with recovery and incubation, empty X-Vivo 15^{TM} droplets were collected from a droplet generator chip lacking the 4-way splitting to yield larger droplets. Three separate droplet populations, orange-stained CAR-T, green-stained RAJI, and Assay Beads were independently generated and combined into an input emulsion in MODE assay assembly. Common to these three populations was 5 nM streptavidin-AlexaFluor 647 (Thermo Fisher) and 5 nM biotinylated α-IFN-γ antibody (R&D catalog no. DY285B). Three levels of Cascade Blue-10 kDa dextran (Thermo Fisher) - 6, 12, and 18 µM, were included in the CAR-T, RAJI and Bead droplets respectively to help with encoding and gating during assembly. The carrier oil used for droplet generation is Novec HFE-7500 (3M) supplemented with 2% wt/wt biocompatible surfactant (Ran Biotechnologies), and during assembly the same carrier oil is used at 0.1% wt/wt surfactant for the bias and spacer oils.

Downstream analysis following MODE assay sorting

Sorted droplet and waste droplet emulsions were broken using an anti-static gun [(1)], and the aqueous phase of each sample removed for sequencing and image analysis. Samples were prepared for single-cell RNA-sequencing using the 10X Chromium Next GEM Single Cell 5' Library Kit (10X catalog no. 1000265) with V(D)J amplification kit (10X catalog no. 1000252) according to the manufacturer's instructions. 5' gene expression and V(D)J-enriched libraries were submitted to Seqmatic for sequencing (https://www.seqmatic.com/). Gene expression and V(D)J libraries were combined in a 4:1 ratio prior to sequencing. Paired-end sequencing was performed on an Illumina NextSeq 550 with a mid-output kit to a depth of 12.5 million reads with a read 1 length of 26 base pairs and read 2 length of 91 base pairs. Sequencing data was aligned to the human GRCh38 reference genome using 10X Genomics Cell

Ranger 3.1.0 pipeline. The FMC63 gene encoding the α CD19 CAR was appended to the GRCh38 reference genome prior to alignment. Effective sequencing depth between the two samples was normalized using the Cell Ranger 3.1.0 aggr tool before comparison. Gene expression analysis was then performed using 10X Genomic's Loupe Browser Version 5.0.0. We initially observed a large number of barcodes containing a low number of UMIs, potentially due to the presence of free RNA from dead Raji cells which had been lysed during incubation in the droplets. To remove these barcodes prior to analysis, we reanalyzed the data using the 10X Genomics Loupe Browser reclustering tool with the following parameters: 4000 UMIs per barcode minimum, 5% mitochondrial UMIs per barcode maximum, dimensionality reduction = 10, and 2D embedding = 30. CD8+ cells were defined as any cells having a $\log_2$ CD8A count ≥ 1 or $\log_2$ CD8B count ≥ 1 or $\log_2$ CD8B2 count ≥ 1. Granzyme B and/or IFN-γ expressing cells were defined as cells having a $\log_2$ GZMB and/or IFNG count ≥ 1.

SI Figures and Tables

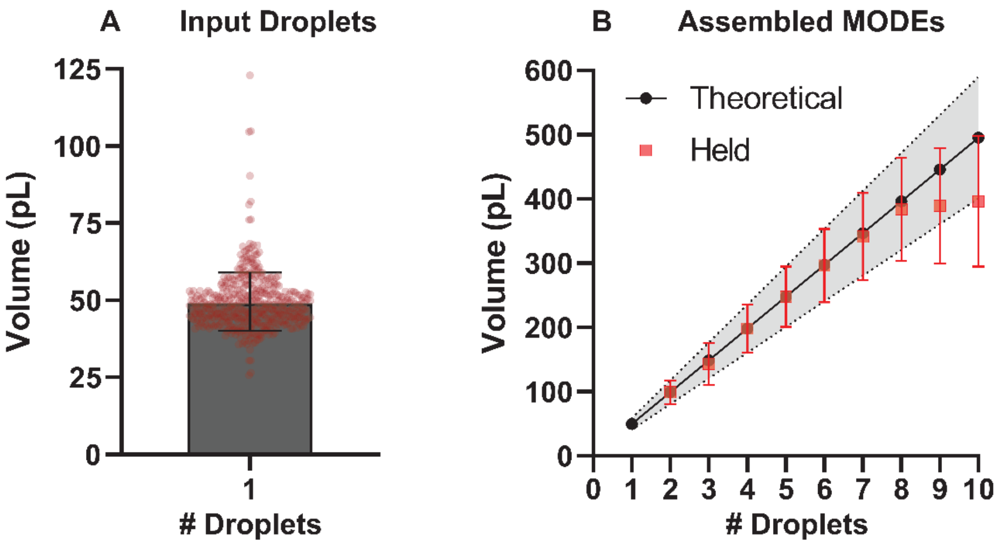

C

	Sorting				Trapping			
Target	no sort	wrong sort	multiple sort	overloaded	no merger	partial merger	release error	No Error Present
2	0.0%	0.0%	2.3%	7.0%	0.0%	0.0%	0.0%	90.7%
3	0.0%	0.0%	14.5%	3.6%	0.0%	5.5%	0.0%	78.2%
4	0.0%	0.0%	9.2%	7.7%	0.0%	12.3%	0.0%	75.4%
5	0.0%	1.2%	7.3%	3.7%	0.0%	13.4%	0.0%	76.8%

Figure SI 1: **Trap volume limits the amount of input droplets that can be assembled into a given MODE.** A) Input droplet volume was calculated via image analysis. B) MODEs ranging from 2 to 10 component droplets were assembled and the number of drops held by the trap was counted in assembly movies. The volume that was held on the trap is compared to the theoretical volume that should be help if every input droplet incorporates into the MODE. C) Common sources of error were counted from frame-by-frame analysis of MODE assembly of 2 through 5 beads (50-80 MODEs analyzed per target MODE size).

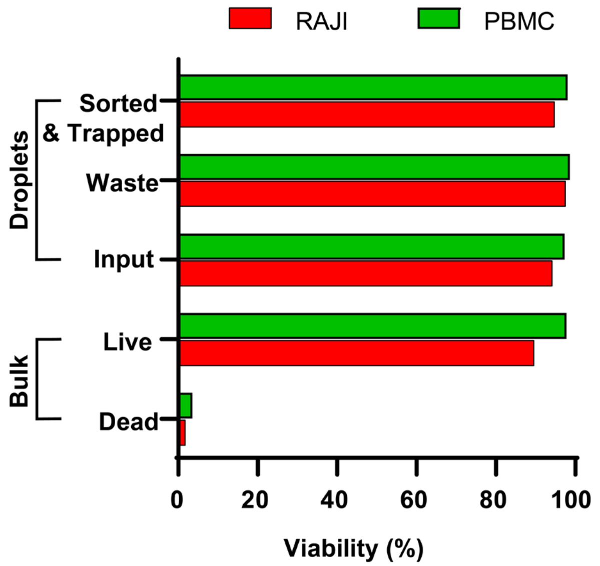

Figure SI 2: **Viability is maintained throughout the process of droplet assembly** Viability of RAJI and PBMC cells for input droplets and collected droplets that were sorted and trapped or sent to waste.

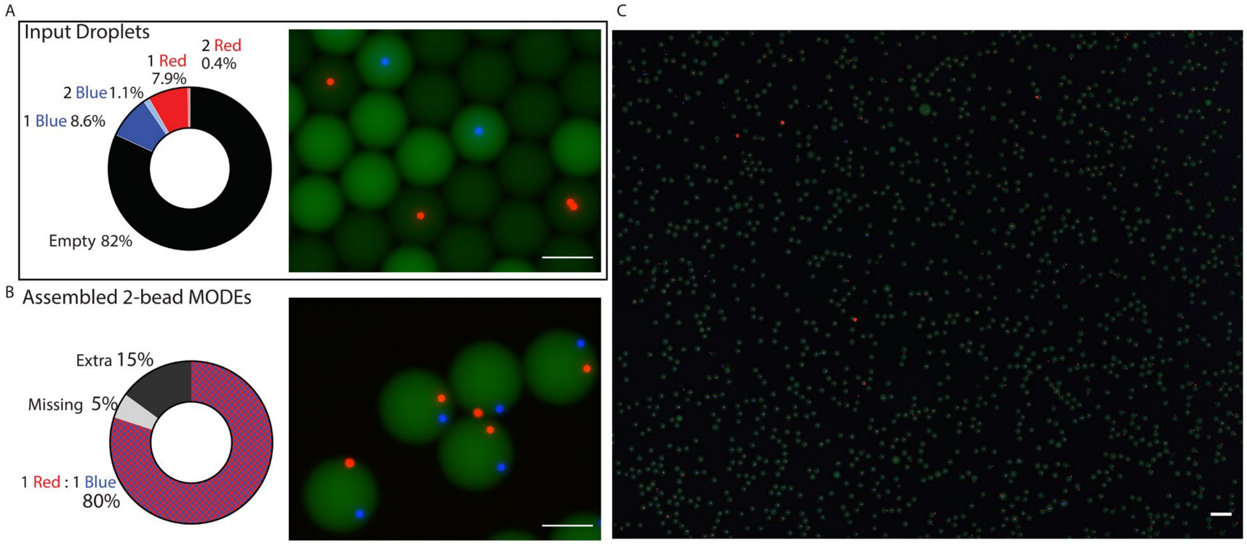

Figure SI 3: **Droplet assembly for 100,000 modes designed to contain 1 red and 1 blue bead.** A) Input droplets were sparsely loaded with either red or blue beads and combined into a single emulsion. B) Accuracy of assembly as assessed by a subset of droplets by fluorescence imaging. C) Zoomed out fluorescent composite image showing a subset of assemble droplets. Scale bars are 50 µm.

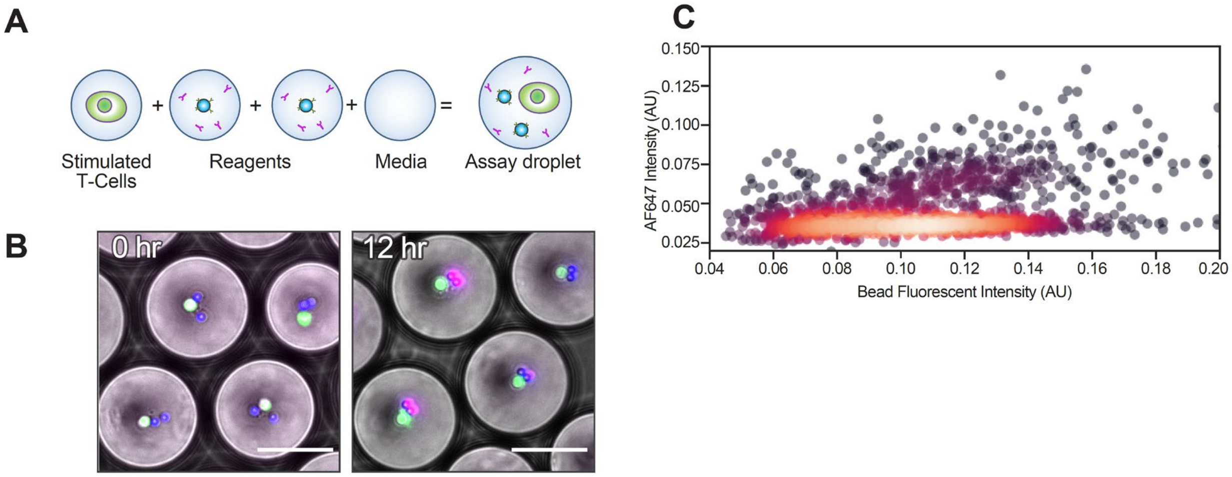

Figure SI 4: **Demonstration of IFN-γ detection from artificially stimulated CAR-T cells.** A) CAR-T cells were stimulated with PMA/ionomycin and assembled into MODE assays. B) Activated cells generated IFN-γ within 12 hours leading to fluorescent relocation onto assay beads. C) Assay hits were detectable on the droplet sorter.

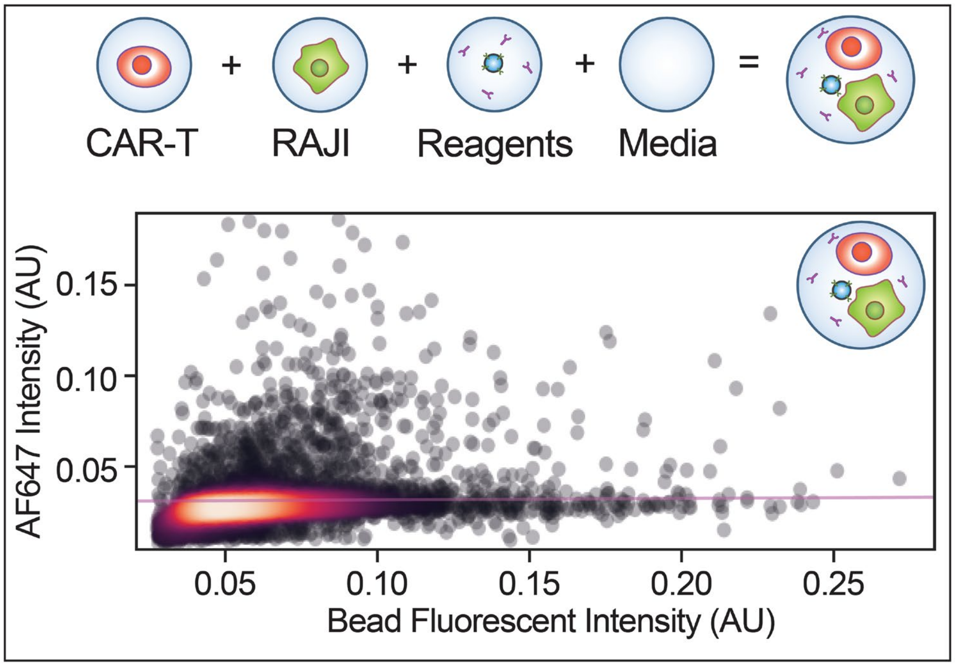

Figure SI 5: **Assembled MODEs were sorted for IFN-γ activation for downstream single-cell RNA sequencing.** CAR-T and RAJI were combined with IFN-γ detection reagents and sorted for signal intensity above a threshold (magenta).

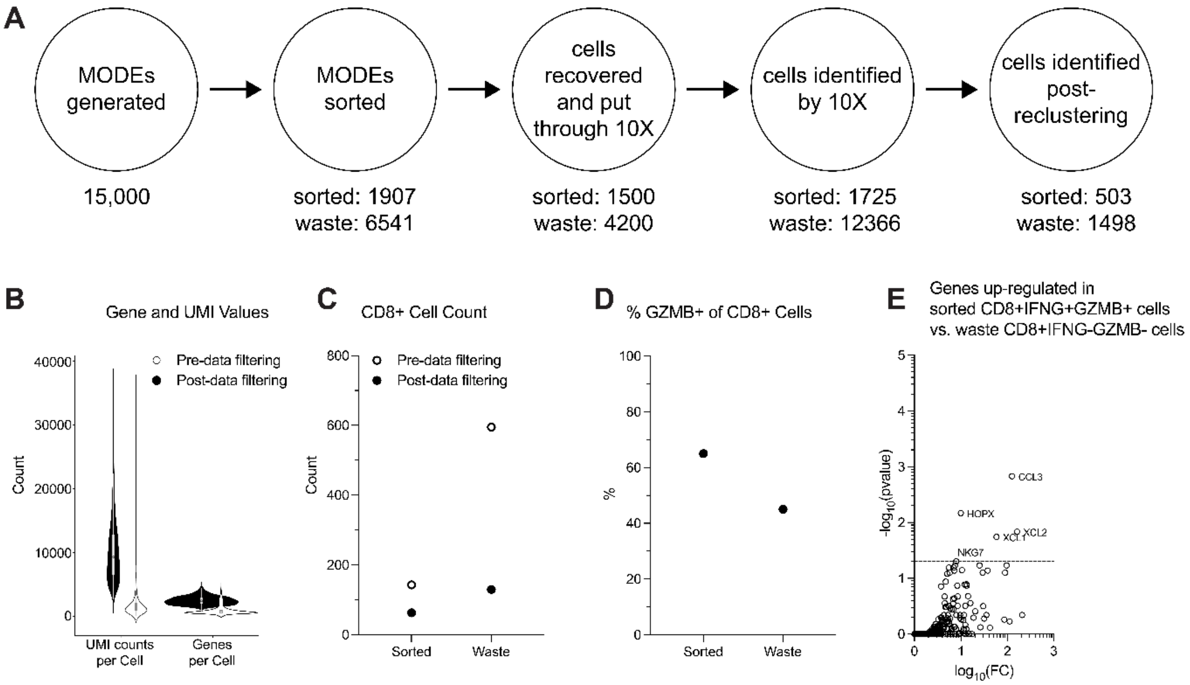

Figure SI 6: A) Workflow for sequencing single cells after MODE generation and sorting. Sorted and waste values under each step represent number of cells. B) Median genes per cell and median UMI count per cell pre- and post-data filtering (reclustering). C) CD8+ cell counts pre- and post-data filtering for sorted and waste populations. D) Percentage of CD8+ cells expressing granzyme B in sorted and waste populations. E) Volcano plot showing genes significantly up-regulated in sorted CD8+ cells expressing IFN-γ and granzyme B relative to waste CD8+ cells not expressing IFN-γ or granzyme B. (Off-axis: GZMB, log10FC = 3.85, -log10(pvalue) = 11.64; IFN-γ, log10FC = 2.88, -log10(pvalue) = 11.64)

Table SI1. Log2 fold-change (FC) and p-values for all genes significantly ($p < 0.05$) up-regulated in the sorted and waste GZMB-expressing sub-populations relative to background (waste cells not expressing GZMB). No significantly down-regulated genes were observed in either population. Strikethrough indicates gene was not detected or log_2 fold-change was not significant.

	Sorted		Waste	
Gene	Log_2 FC	P-Value	Log_2 FC	P-Value
GZMB	15.66	1.51E-36	15.22	1.32E-39
NKG7	5.02	1.99E-10	4.61	1.27E-06
HOPX	4.59	1.43E-08	4.53	6.85E-08
IFNG	6.34	1.43E-08	6.04	4.12E-04
CCL4	6.20	2.64E-07	6.26	1.19E-03
CST7	4.22	2.64E-07	3.99	6.85E-08
CCL5	3.88	6.24E-06	3.54	2.85E-05
TNFRSF9	4.49	2.32E-05		
PHLDA1	4.18	3.69E-05	4.33	6.85E-08
CCL3	5.66	5.20E-05	5.36	1.84E-05
HMOX1	6.36	5.86E-05		
LGALS1	3.47	1.61E-04	3.33	1.01E-04
XCL1	6.87	1.61E-04	6.53	1.84E-05
XCL2	7.04	1.61E-04	7.34	4.67E-04
IL32	3.22	1.70E-04	3.12	6.79E-05
CD3D	3.10	3.95E-04	3.02	1.25E-04
GZMA	5.25	4.86E-04	5.26	2.49E-05
LINC02446	5.34	5.22E-04		
ITM2A	3.44	6.47E-04		
CD44			2.93	7.76E-04
S100A11	3.01	9.53E-04	3.17	2.63E-05
CD3E			2.69	9.87E-04
TNFSF10			3.44	1.00E-03
S100A4			2.78	1.18E-03
CCL1	8.38	1.27E-03		
CSF2	5.11	1.58E-03	4.02	3.17E-02
LGALS3			3.45	2.30E-03
S100A6	2.95	2.66E-03	2.98	2.35E-04
GNLY			6.57	3.16E-03
VIM			2.46	3.80E-03
ANXA2			2.49	4.40E-03
TRAC			2.70	4.56E-03
TNFRSF18			2.94	4.56E-03
LY6E			2.56	5.88E-03
GYPC			2.40	1.02E-02
LCK			2.43	1.02E-02
CD96	2.86	1.06E-02	3.07	1.91E-04
HCST	2.62	1.22E-02	2.51	3.61E-03
TIMP1			3.07	1.29E-02
TRBV10-3	5.34	1.57E-02	3.64	1.77E-02
CYTIP			2.11	1.80E-02
TRBC1			2.36	1.85E-02
HSPB1			2.65	1.98E-02
LAT	2.87	2.07E-02	3.35	6.69E-05
CD7	2.57	2.39E-02	3.22	2.49E-05
SRGN	2.37	2.82E-02	2.28	7.44E-03
SH3BGRL3			2.01	2.90E-02
EMP3			1.97	3.55E-02
GNG2	2.44	3.65E-02		
IL2RA	2.76	4.04E-02	3.74	1.17E-05
RGCC	2.85	4.80E-02	2.46	8.31E-03

Movie SI 1: **Droplet assembly of IFN-γ MODE consisting of a CAR-T cell, RAJI cell, and assay bead and reagents.** Droplets are sorted and merged until all MODE components are included. The drop is released and assembly begins on a new MODE.
